# Extracellular Matrix Remodeling Factors as Markers of Carotid Artery Atherosclerosis

**DOI:** 10.1155/2020/9036157

**Published:** 2020-08-12

**Authors:** Agnieszka Sapa-Wojciechowska, Alina Rak-Pasikowska, Kornel Pormańczuk, Bartłomiej Czapla, Iwona Bil-Lula

**Affiliations:** ^1^Division of Clinical Chemistry and Laboratory Hematology, Department of Medical Laboratory Diagnostics, Faculty of Pharmacy, Wroclaw Medical University, 50-556 Wroclaw, Poland; ^2^Department of Surgery, 4th Military Teaching Hospital in Wroclaw, 50-981 Wroclaw, Poland; ^3^Division of Surgical Specialties, Department of Clinical Nursing, Faculty of Health Science, Wroclaw Medical University, 51-618 Wroclaw, Poland; ^4^Department and Division of Surgical Didactics, Faculty of Medicine, Wroclaw Medical University, 50-369 Wroclaw, Poland

## Abstract

**Materials and Methods:**

20 patients undergoing routine carotid endarterectomy and 40 healthy volunteers were enrolled in this study. MMPs activity and OPG and FN concentrations were measured in atherosclerotic plaques and nonchanged contiguous tissue after homogenization as well as in plasma from patients and reference group. The activity of MMPs was evaluated by gelatin zymography, and the concentration of OPG and FN was assessed by ELISA.

**Results:**

OPG concentration and MMP-9 activity showed differences between plaque and nonchanged tissue; OPG was higher in adjacent tissue (*P*=0.0009), whereas MMP-9 was higher in plaque (proMMP-9 *P*=0.0003; MMP-9 *P* < 0.0001). The OPG plasma concentration and both MMPs plasma activity were higher in patients (OPG *P* < 0.001; proMMP-2 *P*=0.0292; and proMMP-9 *P*=0.0374), while FN plasma concentration was lower in patients than in the reference group (*P*=0.0004). The ROC curves analysis showed the highest AUC for OPG (0.943) with 85.0% sensitivity and 92.1% specificity.

**Conclusions:**

The atherosclerotic plaque and the contiguous artery wall are biochemically different. OPG shows the highest potential to be a marker of advanced carotid atherosclerosis.

## 1. Introduction

Carotid artery stenosis is a serious condition, which is a result of atheromatous plaques formation, and it possesses a major risk factor of stroke and cognitive dysfunction [1, 2]. Endarterectomy is still recommended treatment especially in symptomatic patients, and it should be considered in all patients with degree of stenosis of 50–69% and obviously performed in patients with symptomatic stenosis over 70% [3]. Because the degree of stenosis and the presence of symptoms are not the only elements influencing final clinical outcome, many researches have been conducted to determine if there is a biomarker useful in the proper risk stratification and the choice of patient treatment [4, 5].

**Figure 1:**
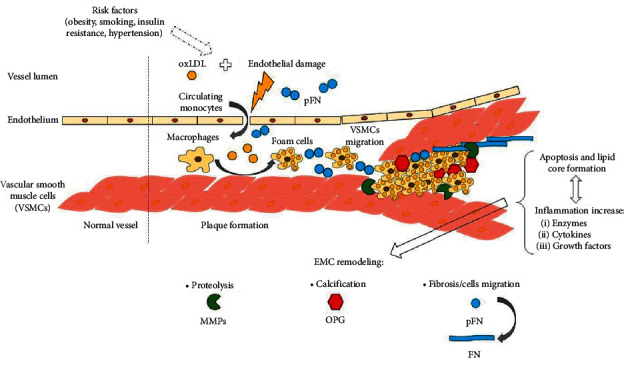
Key processes in atherosclerotic plaque formation.

The atherosclerotic plaque formation is a complex and multifactorial process, whose biology has not been completely understood yet [6]. A key process underlying the formation of atherosclerotic lesion is remodeling of extracellular matrix (ECM), mediated by monocyte-derived macrophages which transform into foam cells as a result of lipids uptake. Apoptosis of foam cells leads to the formation of lipid core and causes further infiltration of inflammatory cells, which secrete cytokines, growth factors, and enzymes (i.e., myeloperoxidase, matrix metalloproteinases (MMPs)) [7, 8]. The crucial aspects of plaque formation are presented in Figure 1.

Local activity and overexpression of MMPs as well as an imbalance in their activation and inhibition by tissue inhibitors of matrix metalloproteinases (TIMPs) are essential in ECM remodeling. MMPs degrade a variety of types of collagen and other proteins like fibronectin, chemokines, and growth factors [9, 10]. Moreover, MMPs are involved in transmigration of vascular smooth muscle cells (VSMCs) between arterial layers, thus influencing plaque thickness and stability. In human elastic arteries, in comparison to the muscular type of systemic arteries, VSMCs are present mostly in tunica media and some of them reside in tunica intima, and their main function is the contraction and dilation of the vasculature [11]. In atherosclerotic lesion, activated VSMCs proliferate and migrate from muscular layer to the core and fibrous cap covering the plaque. They may transform into cells similar to osteoblasts, which release factors regulating calcification. They are also a source of collagen and elastin [11, 12]. Overall, different types of MMPs play different and equivocal roles in the atherosclerosis—some are protective, and some are proatherogenic factors. Generally, MMP-2 is suggested to have a negative association with plaque vulnerability, and MMP-9 is connected with plaque instability [8, 10, 13], but there are also data available which prove that MMP-9 is primarily involved in vascular repair and collagen organization causing plaque stabilization [10, 14].

Calcification is another ambiguous phenomenon, which is usually associated with plaque stability, but on the other hand, calcified plaques are brittle and more susceptible to damage. It depends mostly on the artery wall layer, which is affected, because calcification of intima causes plaque vulnerability and media-arterial stiffness [11, 12]. One of the proteins involved in the calcification is osteoprotegerin (OPG). OPG is a protein produced by different types of cells like osteoblasts, monocytes, endothelial cells, neutrophils, fibroblasts, and smooth muscle cells. As a member of tumor necrosis factor superfamily, it is responsible for proper bone formation as it is an osteoclastogenesis inhibitor via receptor activator of NF-*κ*B ligand (RANKL)/receptor activator of NF-*κ*B (RANK)/OPG system [15]. The role of OPG in plaque formation is unclear; since it has been reported that OPG prevents calcification by anti-inflammatory action, it stimulates angiogenic signaling pathways, but it also diminishes endothelium proliferation enabling migration of inflammatory cells, and it inhibits their apoptosis. Moreover, OPG enhances expression of adhesins and local synthesis of MMPs, which can degrade the fibrous cap of a plaque [16, 17].

Another aspect of biological changes in the atherosclerosis in the context of plaque stability is deposition of fibronectin (FN) in arterial wall. FN is a matrix glycoprotein produced by hepatocytes, macrophages, VSMCs and osteoblasts, which regulates cells adhesion, migration, and proliferation [18]. FN can be produced in the plaque or absorbed from plasma into atheromatous plaque [19]. Fibrosis is a process that stabilizes the plaque, but fibronectin plays an important role in endothelial permeability and sustaining inflammatory state [20]. In mice model, plasma fibronectin (pFN) deficiency affected arterial walls interfering with fibrous cap formation and thus increasing plaque vulnerability [21]. The pFN concentration may impact the evolution of the plaque, but there are discrepancies in the results of different studies, showing that the mechanism of FN incorporation in atherosclerotic plaque is not completely understood and may be affected by many factors [22].

Advanced possibilities in protein analysis enable searching for the new markers of atherosclerosis. The analysis of proteome and secretome of unstable plaques revealed numerous proteins of diagnostic potential [23, 24]. On the basis of the secretome, we chose important proteins representing different but dependent aspects of ECM remodeling that are released by plaques. In this study, we evaluated the tissue content of MMP-2 and MMP-9, osteoprotegerin, and fibronectin in atheromatous artery wall and the plasma level of the mentioned parameters. We aimed to elucidate if the local changes of above proteins have a systemic reflection and to assess their clinical utility in the patients with carotid atherosclerosis by the comparison with reference group.

## 2. Materials and Methods

### 2.1. Subjects and Clinical Material

**Figure 2:**
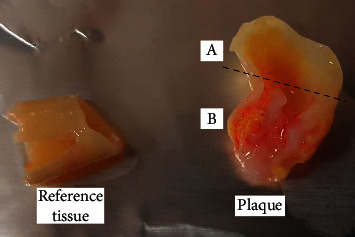
Representative tissue fragments. The fragment A above the dotted line was separated and removed. Fragment B was used as atherosclerotic plaque.

Atherosclerotic plaques were obtained from 20 patients undergoing routine carotid endarterectomy at the Department of Vascular Surgery of the 4th Military Teaching Hospital in Wroclaw. Patients demographics is presented in Table 1. Immediately after collection, artery fragments were submerged in physiological saline to remove blood and then transported at 4°C in saline within 3 hrs to the laboratory, where each material was divided into two sections under eye control—the thick, ulcerating, hemorrhagic, and calcified part of the tissue with the lipid deposits (further called “plaque”) and the margins of the specimen representing the more stable part of the arterial wall (further called “reference tissue”), which did not exhibit pronounced symptoms of atherosclerotic process. Representative tissue fragments after dissection are presented in Figure 2. All fragments were promptly frozen in liquid nitrogen to prevent protein degradation and then stored at −80°C until further preparation.

All patients and 40 volunteers comprising reference group underwent basic laboratory studies including whole blood count, triglycerides, total cholesterol, high-density lipoprotein cholesterol (HDL-C), low-density lipoprotein cholesterol (LDL-C), and creatinine. To determine the MMPs activity and OPG and FN concentration, citrated blood samples were collected from each patient before surgery and from volunteers who are also characterized in Table 1. Plasma was obtained by a standard protocol, and samples were stored at −80°C until use.

The study was conducted in accordance with the Declaration of Helsinki and was approved by the Institutional Review Board and by Bioethics Committee of Wroclaw Medical University (No. KB729/2017). The written consent was taken from each participant of the study.

### 2.2. Tissue Homogenates Preparation

Prior to biochemical assays, tissue samples were homogenized in liquid nitrogen using porcelain mortar and a pestle. The proper amount of powder was transferred into fresh tube and cells were disrupted with homogenization buffer (150 mM NaCl, 50 mM Tris-HCl pH 7,4, 1% Triton® X-100, Protease Inhibitor Cocktail without ethylenediaminetetraacetic acid set III (Sigma)) in the proportion of 1 + 4 (w/v) to prepare 20% homogenates. Tissue suspensions were further homogenized by Pellet Pestle® Motor (Kimble Kontes); each sample underwent 4 cycles of homogenization for 10 seconds on ice and then centrifuged at 14,000 × *g* for 5 min. Supernatants were stored at −80°C and thawed prior to analysis.

### 2.3. Gelatin Zymography

**Figure 3:**
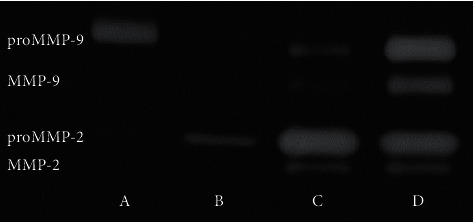
Representative zymogram of different materials from one patient. A–capillary blood standard; B–plasma; C–reference tissue; and D–plaque.

The activity of MMPs was determined in citrated plasma samples and homogenates by gelatin zymography according to the procedure described by Heussen and Dowdle [25] with modifications. Zymography enables the detection of both latent and active forms of MMP-2 and MMP-9, which can be distinguished on the basis of their molecular weight. Prior to zymography, protein content was determined in tissue homogenates and diluted plasma samples by Protein Assay Dye Reagent Concentrate (Bio-Rad). All samples were further diluted to achieve 20 *µ*g of protein in 20 *µ*L and mixed with the proper amount of 4*x* Laemmli Sample Buffer (Bio-Rad). Samples were loaded in 7.5% polyacrylamide gels copolymerized with 2 mg/ml pork gelatin (Sigma) containing 0.1% SDS. After electrophoresis (120 V, 4°C), gels were washed three times for 20 min in 2.5% Triton X-100 and then incubated in development buffer (50 mmol/l Tris-HCl, pH 7.5, 200 mmol/l NaCl, 5 mmol/l CaCl_2_, and 0.05% NaN_3_) at 37°C, for 18 hours. The staining was performed in 0.5% Coomassie Brilliant Blue R-250, 30% methanol, and 10% acetic acid for 2 hours, and destaining was performed in 30% methanol, 10% acetic acid until the clear bands on the blue background were distinctly visible. Gels were scanned using GS-800 Calibrated Densitometer with Quantity One v. 4.6.9 software (Bio-Rad), and the relative MMPs activity was determined and expressed in arbitrary units (AU) per milligram of total protein in a sample. Healthy volunteer's capillary blood served as a standard of MMPs activity according to Makowski and Ramsby [26]. Representative zymogram of different materials from the same patient is presented in Figure 3.

### 2.4. Osteoprotegerin and Fibronectin Determination by Enzyme-Linked Immunosorbent Assay (ELISA)

Osteoprotegerin concentration was measured by ELISA Kit for Human Tumor necrosis factor receptor superfamily member 11B (EIAab®, Cat. No. E0108 h). Homogenates were diluted 500-fold before measurement, and the plasma was not diluted. Fibronectin concentration was determined in all samples by Human Fibronectin ELISA kit (EIAab®, Cat. No. E0037 h). Plasma samples were diluted 100,000-fold, and tissue samples were diluted 5000-fold before measurement. All samples were run in duplicate, and the concentration was calculated on the basis of the calibration curve prepared according to the manufacturer's protocol. The arithmetic mean of two readings was taken for statistical analysis. Plates were read with UV/Vis-Multiskan GO spectrophotometer (Thermo Scientific).

### 2.5. Statistical Analysis

Statistical analysis was performed by GraphPad Prism, version 6.07 (GraphPad Software Inc.) and MedCalc Statistical Software, version 19.2.1 (MedCalc Software Ltd., Ostend, Belgium; https://www.medcalc.org; 2020). D'Agostino–Pearson test was used to determine data distribution. Chi-squared test was used for comparison of patients and reference group characteristics and comorbidities. Tests used for data comparison included *t*-test, Welch's test or Mann–Whitney test, depending on the normality of data distribution and the equality of variances to analyse results in plasma between groups; paired-samples *t*-test or Wilcoxon's test for comparison of data in different materials within the same patients. Pearson's correlation coefficient was used to assess the correlation between OPG, FN, MMPs and BMI, age, and chosen laboratory test results, as well as the correlation between tissue content and plasma level of the markers. To assess the diagnostic value of the markers, ROC curves analysis was performed including comparison of area under the curve (AUC) based on methodology by DeLong et al. [27] available in MedCalc. Analysis of binary logistic regression in four models including each of the markers was performed to evaluate the contribution of selected additional atherosclerosis risk factors in carotid artery atherosclerosis. Logistic regression model was also used to calculate the predicted probabilities for classical risk markers of atherosclerosis (i.e., total cholesterol, hypertension, male sex, and smoking), which were also used in ROC curves comparison.

## 3. Results and Discussion

**Figure 4:**
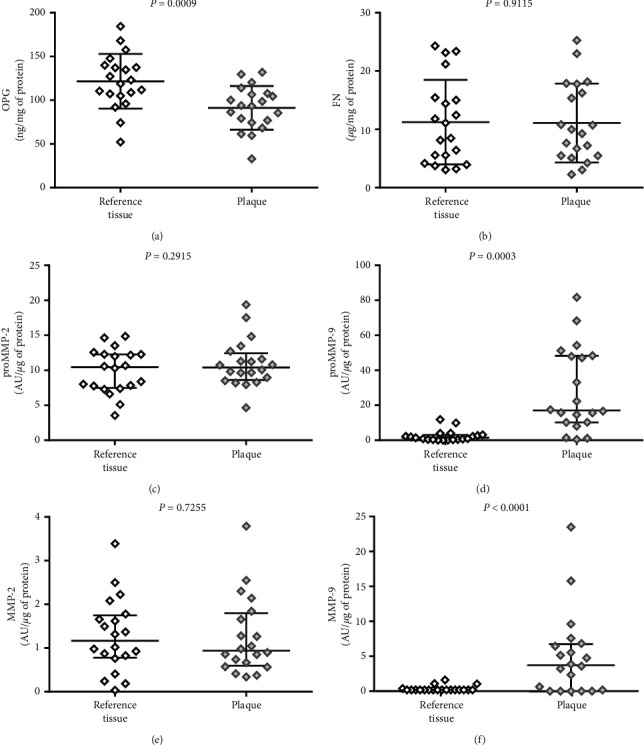
Comparison of (a) osteoprotegerin (OPG), (b) fibronectin (FN), (c) matrix metalloproteinase 2 proenzyme (proMMP-2), (d) matrix metalloproteinase 9 proenzyme (proMMP-9), (e) matrix metalloproteinase 2 active form (MMP-2), and (f) matrix metalloproteinase 9 active form (MMP-9) in atherosclerotic plaque and reference tissue, from the same patients. The middle lines represent the median, and the whiskers extend from the 25th to the 75th percentiles.

Since matrix metalloproteinases, osteoprotegerin, and fibronectin have been analysed in the past years as proteins involved in extracellular matrix remodeling, they could be used as potential indicators of carotid artery atherosclerosis [5] or plaque instability [4, 10] and markers of risk stratification of acute complications like stroke or cardiac infarction [28–30]. In this study, we evaluated the tissue content of OPG, FN, and MMPs in the area of human atherosclerotic plaque and their plasma concentration or activity. This is the first comparison of OPG, FN, and MMPs in tissue materials from the same patient which revealed that atherosclerotic plaque and contiguous arterial wall tissue are biochemically different. Results of this analysis are presented in Figure 4.

Differences observed by us were sometimes surprising, as we discovered higher amount of osteoprotegerin in the reference tissue than in the area of plaque itself (*P*=0.0009). All of the analysed plaques presented macroscopic features of vulnerability, but they were strongly calcified. OPG was previously described as an inhibitor of pathological vascular calcification, but the disturbances in RANKL/RANK/OPG system are important in plaque vulnerability [16, 17]. Lower amount of OPG in the central part of the plaque may be responsible for its strong calcification. In the study by Hakimi et al. [31], the OPG level estimated by immunohistochemical staining was the highest in the marginal zone of the calcified atherosclerotic plaque, not in the central part, and correlated with the presence of symptoms in the patients. We hypothesize that the low level of OPG in the core of the lesion in our study may be a result of OPG consumption as a regulator of calcification and apoptosis processes and/or its degradation by locally enhanced MMPs, mostly MMP-9 whose activity was very high in the plaques (proMMP-9 *P*=0.0003; MMP-9 *P* < 0.0001). High activity of proenzyme is a result of enhanced gene expression, and high activity of enzyme may be a result of both its overexpression and altered inhibition by TIMPs. The extremely high activity of MMP-9 in the vulnerable plaques is consistent with publications indicating the inflammatory source of MMP-9 in atherosclerotic lesions and its destabilizing effect [4, 32]. We did not observe similar differences for any form of MMP-2 activity (proMMP-2 *P*=0.2951; MMP-2 *P*=0.7255). Its role in the plaque formation is dual because it can lead to progression and destabilization of the plaque and, on the other hand, can cause the healing effects mainly by regulating VSMCs migration, whose effects depend mostly on the dominant form of MMP-2 (active or proenzyme) and the particular activation pathway [10, 32]. Heo et al. [28] observed the higher amounts of both MMPs in unstable plaques which may suggest that reference tissue in our study was also affected by matrix remodeling in spite of lack of macroscopic changes, but these discrepancies may also be an effect of the different assays used for MMPs detection, as we used gelatin zymography which shows the real activity of the enzymes, not their immunohistochemical expression. The distinction between different phenotype of MMPs activity as well as OPG level in vulnerable plaques is important in the context of possible innovative treatment with selective inhibitors [10, 16, 33] or detection of vulnerable plaques by specific molecular probes [34, 35].

Another interesting observation in this study was the equal amount of FN in the atherosclerotic plaque and the reference tissue. Fibronectin is an important regulatory protein of ECM and its deposition in arterial wall was previously described as a process being partially responsible for fibrosis and plaque stability [21, 36, 37]. The tissue content of FN was associated mostly with the type of the lesion–fibrous plaques poor in lipids containing higher levels of FN, whereas lipid-rich necrotic plaques had lower levels of FN [38]. Tissue deposition of FN called fibrillogenesis is a cell-mediated process that contributes to regulation of local inflammation [18, 20]. The lipid core of the plaques lacks the ability of FN binding, and FN may be also degraded by MMPs in unstable atherosclerotic plaques, so the high activity of MMP-9 observed in our study may contribute to the equal amounts of FN in both examined types of tissue fragments (*P*=0.9115). Moreover, these results may suggest that the remodeling process affects not only the plaque area but the artery wall in general (as atherosclerosis is a systemic disease).

Nevertheless, none of the parameters tissue level correlated with its plasma concentration or activity. The results of correlation analysis are presented in Table 2. The lack of correlation for reference tissue is not surprising, but no correlation of the parameters in plasma with their plaque content suggests that local tissue changes are not indirectly linked to plasma level of these proteins and more complex processes underlie observed plasma differences between study and reference group.

Due to the limited sample size, we performed the preliminary assessment of the clinical utility of OPG, FN, MMP-2, and MMP-9 as atherosclerosis markers and it was made on the basis of comparison of the markers in the plasma of patients undergoing endarterectomy and reference group. Reference group was chosen to represent relatively low risk of atherosclerosis. Thus, the composition of the reference group was different from the study group primarily in the age context (*P* < 0.0001), so we determined the validity of the reference group by calculation of correlation between age and the level of OPG, FN, and MMPs in the plasma. We did not observe relevant correlation between age and any of the assessed parameters (proMMP-2 *R* = 0.1206, *P*=0.4585; proMMP-9 *R* = 0.3393, *P*=0.0619; OPG *R* = 0.2556, *P*=0.1114; and pFN *R* = 0.03006, *P*=0.8539), as well as no correlation of BMI with any of them (proMMP-2 *R* = −0.1249, *P*=0.4485; proMMP-9 *R* = 0.2805, *P*=0.1200; OPG *R* = 0.1056, *P*=0.5222; and pFN *R* = −0.003664, *P*=0.9823). The studied group of women was too small to assess the correlation with sex, but available publications usually show no correlation [39, 40]. Although our results indicate that there is no relationship of age and BMI with evaluated plasma atherosclerosis markers, the literature data is not consistent in this matter, especially concerning OPG. Some of the publications confirm no connection of OPG with age [39, 41], but others show positive correlation between these parameters [42, 43]. Hence, we performed binary logistic regression analysis to evaluate the influence of other important parameters along with matrix remodeling markers on the risk of atherosclerosis complications requiring carotid endarterectomy. Results of this analysis are presented in Table 3. Multifactorial analysis of four logistic models revealed that hypertension and OPG have significant impact on the risk of unstable atherosclerotic plaques presence. Other evaluated markers (i.e., pFN, proMMP-2, and proMMP-9) did not significantly contribute to the prediction of atherosclerosis in evaluated models. However, in the model containing FN, both hypertension and smoking were important, and hypertension and white blood cells count for proMMP-2 and proMMP-9 were factors influencing the necessity of endarterectomy. Hypertension is a well-known risk factor, and it was common in our patients, but all of them received hypotensive therapy; thus, its influence on the results may be lower with the proper medical control.

**Figure 5:**
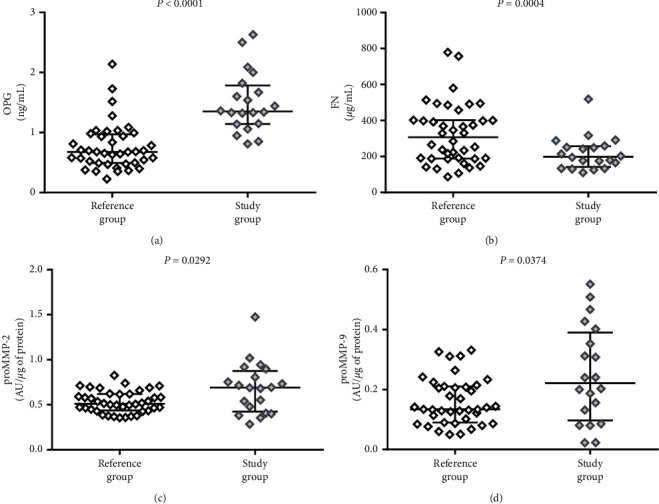
Comparison of (a) osteoprotegerin (OPG), (b) fibronectin (FN), (c) matrix metalloproteinase 2 proenzyme (proMMP-2), and (d) matrix metalloproteinase 9 proenzyme (proMMP-9) in plasma of patients (study group) and reference group. The middle lines represent the median, and the whiskers extend from the 25th to the 75th percentiles.

**Figure 6:**
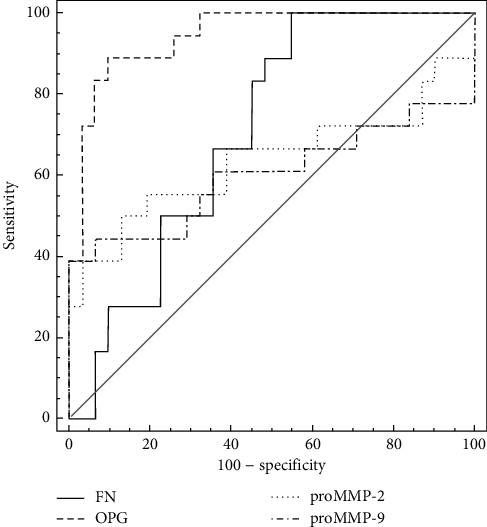
ROC curve comparison for single markers. AUC for OPG is 0.943 (*P* < 0.001), with associated criterion >1.0349 ng/ml; sensitivity 85.0%, specificity 92.1%. AUC for FN is 0.712 for ≤316.9966 *µ*g/ml; sensitivity 100%, specificity 47.4%. AUC for proMMP-2 is 0.676 for activity >0.6897 AU/mg; sensitivity 55.0%, specificity 87.5%. AUC for proMMP-9 is 0.625, with associated criterion >0.2637 AU/mg, sensitivity 42.1%, and specificity 100.0%.

**Figure 7:**
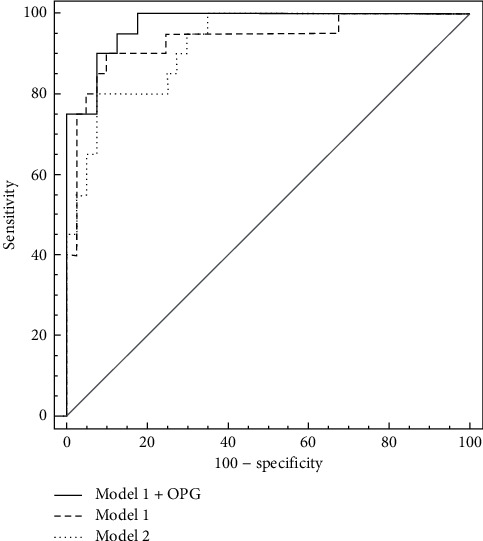
ROC curve comparison for combined models. Model 1 consists of hypertension, current smoking, male sex, and total cholesterol, whereas model 2 consists of OPG, FN, proMMP-2, and proMMP-9. AUC for model 1 is 0.934 (*P* < 0.0001, sensitivity 90.0%, and specificity 90.0%), for model 1 + OPG is 0.974 (*P* < 0.0001, sensitivity 90.0%, and specificity 92.5%), and for model 2 it is 0.922 (*P* < 0.0001, sensitivity 80.0%, and specificity 92.5%).

The comparison of plasma levels of the parameters between the study and reference group revealed that OPG concentration and both MMPs activity are higher in patients with confirmed atherosclerosis of carotid arteries, whereas pFN is lower in patients than in reference group. Results are presented in Figure 5. The higher OPG level in the study group (*P* < 0.0001) along with its described above higher level in the reference tissue fragments suggests that OPG in plasma of patients with symptomatic atherosclerosis may be a result of deep remodeling in the wall of arteries, outreaching the atherosclerotic lesion area. OPG is a parameter that had been extensively investigated in the past years, and its diagnostic potential was documented as a marker of aortic plaque presence and calcification progress [44], plaque instability [17], and distinction between symptomatic and asymptomatic patients [5] and as an independent/additional risk factor in patients with advanced atherosclerosis [45]. Despite the promising results of these studies, osteoprotegerin has not been implemented in routine use so far, so every new study showing its usefulness in the diagnosis of atherosclerosis is valuable. Estimation of ROC curve for OPG revealed relatively high diagnostic value for advanced atherosclerosis in symptomatic patients *versus* healthy subjects (AUC = 0.9434). The comparison of ROC curves for tested markers and combined model including several already known risk factors of atherosclerosis is presented in Figures 6 and 7. High AUC for OPG is consistent with the results of logistic regression analysis. Although the AUC for classical factors of atherosclerosis (i.e., hypertension, smoking, male sex, and total cholesterol) was very high (AUC = 0.934), the addition of OPG seemed to increase AUC to 0.974, yet the difference between areas was not statistically significant (*P*=0.1709).

Higher proMMP-9 and proMMP-2 activity level in study group plasma samples (proMMP-9 *P* = 0.0374; proMMP-2 *P* = 0.0292) is probably a result of chronic inflammation that is related to atherosclerosis, and the high MMP-9 activity is consistent with its high level in the plaque. MMPs, especially MMP-9, are factors with confirmed role in atherosclerosis development and progression, although their clinical utility is still doubtful no matter if it concerns enzymatic activity or protein concentration [4, 5, 46]. However, there are some studies which confirm their ability to predict the presence of unstable plaque in symptomatic patients [47] or restenosis of carotid artery [48, 49]. Area under the ROC curve for MMPs in our study was significantly lower for both MMPs in comparison to osteoprotegerin, although it was relatively high (AUC = 0.6763 for proMMP-2 and AUC = 0.6254 for proMMP-9), showing their clinical potential as additional markers of concomitant inflammation in atherosclerosis, but they are not sensitive enough to be applied as single markers of carotid artery atherosclerosis. On the contrary, plasma fibronectin concentration in the study group was lower than that in the reference group (*P* = 0.0004). This observation is consistent with publications indicating that pFN in atherosclerosis is cleared from plasma by its incorporation in affected arterial wall or aggregation of unstable FN, and thus, low circulating FN may be a marker of atherosclerosis [50]. Moreover, pFN is a protein built-up into thrombus formation, so in patients with unstable plaque, it may be consumed as a result of thrombotic complications [22]. Nonetheless, some studies indicate the higher plasma fibronectin concentration in patients at risk of coronary artery disease [30, 51, 52], but there are also data that confirm the clinical utility of EDA-FN (fibronectin containing extra domain A) but no differences in pFN in citrated plasma of patients with coronary artery disease versus healthy control group [29, 53]. AUC for pFN in our study was 0.712, thus indicating that lower concentration in patients may be connected with advanced atherosclerosis although this parameter lacks specificity. Nevertheless, even the combination of all tested markers (Figure 7) was not more efficient in the prediction of the presence of unstable atherosclerotic plaques in carotid arteries (AUC = 0.922, *P* < 0.001) than the set of classical markers or osteoprotegerin itself, as the difference between AUC was not significant (*P* = 0.8296).

We are aware of the limitations of the study depending mostly on the relatively small size of the study group, which was very homogenous in the aspects of age, sex, and ethnicity. Some of the comorbidities like hypertension, smoking, or administered drugs can influence tested parameters. Estimated very high diagnostic value of osteoprotegerin may be biased by these factors, so the results should be verified on more numerous groups before implementing into routine use.

## 4. Conclusions

Atherosclerotic plaque and the contiguous artery show biochemical differences in osteoprotegerin concentration as well as MMP-9 activity, but there are no differences in both MMP-2 activity and fibronectin concentration. OPG has the best potential to be a marker of carotid atherosclerosis; however, it does not come directly from the necrotic core of the plaque. High plasma activity of MMP-2 and MMP-9 as well as low pFN level have questionable diagnostic value as additional markers of unstable carotid plaque. Analysis of tissue content of these markers and the interrelation between them as extracellular matrix contents provides an additional insight into the biology of atherosclerosis and future diagnostic and therapeutic potential.

## Figures and Tables

**Table 1:** Demographics and comorbidities of the study and reference group.

Characteristics	Study group (*N* = 20)	Reference group (*N* = 40)	Significance level
Age (years)*∗*	70.6 (63.5–78.5)	45.8 (38.0–53.6)	*P* < 0.0001#
Sex (F/M)	5/15	20/20	*P*=0.0663
BMI (kg/m^2^)*∗*	26.89 (23.48–30.30)	24.1 (22.96–26.69)	*P*=0.0496#
Smokers at present (yes/no)	8/12	5/35	*P* < 0.0001#
Arterial hypertension (yes/no)	15/5	4/36	*P* < 0.0001#
Diabetes mellitus or prediabetic condition (yes/no)	10/10	0/40	*P* < 0.0001#
Degree of stenosis of ICA undergoing endarterectomy:			N.A.
60–70%	8	N.A.	
71–84%	4	N.A.	
85–95%	7	N.A.	
>95%	1	N.A.	
Single-vessel atherosclerosis/multiple-vessel atherosclerosis	6/14	N.A.	N.A.
Stroke or TIA in the past (yes/no)	8/12	0/40	*P* < 0.0001#
Neurological symptoms of arterial occlusion within a year before surgery (symptomatic/asymptomatic)	15/5	N.A.	N.A.
Myocardial infarction in the past or ischemic coronary disease (yes/no)	7/13	0/40	*P* < 0.0001#
Medications (grouped) % of the group:			
Calcium channel blockers	40	5	*P*=0.0007#
Beta-blockers	30	2.5	*P*=0.0019#
ACE inhibitors	50	5	*P* < 0.0001#
Diuretics	30	2.5	*P*=0.0019#
Statins	80	2.5	*P* < 0.0001#
Acetylsalicylic acid or other antiplatelet drugs	85	2.5	*P* < 0.0001#
Heparin (LMWH)	15	0	*P*=0.0127#
Insulin	15	0	*P*=0.0127#
Oral hypoglycemic medications	60	0	*P* < 0.0001#
Laboratory test results^*∗*^			
White blood count (G/l)	8.5 (7.85–10.45)	5.35 (4.85–6.05)	*P*=0.0001#
Platelet count (G/l)	222 (188–332)	219 (185–244)	*P*=0.0674
Total cholesterol (mmol/l)	3.55 (2.78–4.48)	4.51 (3.77–5.23)	*P*=0.0518
HDL-C (mmol/l)	1.26 (0.98–1.55)	1.58 (1.35–1.81)	*P*=0.0008#
HDL-C (mmol/l) F	1.37 (1.06–1.68)	1.73 (1.60–2.12)	*P*=0.1044
HDL-C (mmol/l) M	1.22 (1.04–1.41)	1.49 (1.24–1.73)	*P*=0.0082#
LDL-C (mmol/l)	1.90 (1.26–2.51)	2.49 (1.81–3.16)	*P*=0.1608
Triglycerides (mmol/l)	1.06 (0.62–1.50)	0.81 (0.60–1.10)	*P*=0.2821
Creatinine (*µ*mol/l)	72.3 (60.9–85.6)	78.5 (66.2–90.8)	*P*=0.9800
Creatinine (*µ*mol/l) F	56.4 (54.2–61.9)	71.4 (62.6–80.3)	*P*=0.0032#
Creatinine (*µ*mol/l) M	79.4 (69.7–90.8)	86.4 (75.0–97.9)	*P*=0.9832

^*∗*^Data are presented as mean ± SD for parameter showing normal distribution or as median ± interquartile range when the distribution shows discrepancies from normal. #*P* < 0.05 was considered statistically significant. BMI: body mass index; ICA: internal carotid artery; N.A.: not applicable; TIA: transient ischemic attack; ACE: angiotensin-converting enzyme; LMWH: low-molecular-weight heparin; HDL-C: high-density lipoprotein cholesterol; F: female; M: male; LDL-C: low-density lipoprotein cholesterol; and SD: standard deviation.

**Table 2:** Correlations between the tissue fragments and plasma content of OPG, FN, proMMP-2, and proMMP-9.

Marker	Reference tissue vs. plasma		Plaque vs. plasma	
	*R* ^2^	*P*	*R* ^2^	*P*
OPG	0.07647	0.2379	0.00972	0.6792
FN	0.01620	0.5928	0.01960	0.5561
proMMP-2	0.16240	0.0781	0.01074	0.6637
proMMP-9	0.00317	0.8135	0.01833	0.5693

**Table 3:** Multiple-factor binary logistic regression analysis of an influence of chosen independent variables including plasma level of OPG, pFN, proMMP-2, and proMMP-9 as predictors of the risk of endarterectomy.

Independent variables	B	S.E.	Wald	*P*	Odds ratio
OPG	3.47248	1.30591	7.0706	**0.0078**#	32.2166
Hypertension	3.67218	1.15123	10.1747	**0.0014**#	39.3375
Sex (male)	0.20183	1.00707	0.04017	0.8412	1.2236
Smoking (current)	1.75103	1.17237	2.2308	0.1353	5.7605

pFN	-0.0063167	0.0036775	2.9504	0.0859	0.9937
Hypertension	3.55459	1.21174	8.6051	**0.0034**#	34.9736
Sex (male)	1.05097	1.10861	0.8987	0.3431	2.8604
Smoking (current)	2.83889	1.31857	4.6355	**0.0313**#	17.0968

proMMP-2	5.66930	3.41510	2.7558	0.0969	289.8325
Hypertension	2.65427	1.24455	4.5485	**0.0329**#	14.2147
Sex (male)	0.15236	1.20924	0.01587	0.8997	1.1646
Smoking (current)	3.02960	1.76443	2.9482	0.0860	20.6890
WBC	1.18472	0.39212	9.1284	**0.0025**#	3.2698

proMMP-9	0.41722	2.60737	0.02561	0.8729	1.5177
Hypertension	3.16472	1.15277	7.5368	**0.0060**#	23.6821
Sex (male)	0.79772	1.15184	0.4796	0.4886	2.2205
Smoking (current)	2.40338	1.54465	2.4210	0.1197	11.0605
WBC	0.93230	0.33800	7.6080	**0.0058**#	2.5403

^#^ *P* < 0.05 was considered statistically significant. B: regression coefficient; S.E.: standard error; OPG: osteoprotegerin; pFN: plasma fibronectin; proMMP-2: matrix metalloproteinase 2 proenzyme; proMMP-9: matrix metalloproteinase 9 proenzyme; and WBC: white blood cells.

## Data Availability

The data used to support the findings of this study are mostly confidential, so they cannot be made freely available. Requests for access to the data should be addressed to the corresponding author (agnieszka.sapa-wojciechowska@umed.wroc.pl).
